# Personal

**Published:** 1915-12

**Authors:** 


### PERSONAL.

**Announcement of removal, travel, and other matters of interest are requested. Please report errors in the listing of any physician in the State and other directories, that we may co-operate with the State Society in securing a correct list.**

Dr. Edgar R. McGuire of Buffalo, visited Rochester, Minn., early in November, returning intact.

Dr. John Hamilton of Elmira spent last week visiting the clinics in Buffalo.

Dr. E. H. Noble was operated upon the first of the month.

Dr. J. L. Kinner will read a paper on Focal Infection at the Elmira Clinical Society on November 23, 1915.

The report of the Buffalo Ward at the American Ambulance at Paris published in our November issue, was secured through the kindness of Dr. Charles VanBergen of Buffalo.

Dr. Thomas P. C. Barnard of North Tonawanda recently presented a handsome silver emblem to the Tonawanda Lodge No. 247 F. & A. M. He joined this lodge 21 years ago but is now a member of Sutherland Lodge of North Tonawanda.

Dr. Arthur S. Bugbee of Medina was married to Miss Mary Helen Wilds October 12.

Dr. Robert A. Toms of Kenmore has been elected Supervisor of the town of Tonawanda.

Dr. Carlton Wertz, who has been an interne at the Deaconess Hospital, has located at 1440 East Ferry Street, formerly occupied by Dr. C. P. Eller, who has moved from Buffalo.

Dr. A. J. Harris of Buffalo announces his removal to 442 East Utica Street.

---

Dr. S. Y. Howell of Buffalo has recently returned from a trip to Chicago and Rochester, Minn.

---

Dr. Irving M. Snow of Buffalo has moved to 737 Delaware Avenue.

---

Dr. W. D. Preston of Attica has bought and will occupy the residence of Wm. D. Reynolds, on Main Street.

---

Dr. Evelyn F. Frisbie has been elected president of the New Mexico Medical Society.

---

Dr. Charles J. Barone of Buffalo has been appointed to the resident staff of the Elizabeth Steel Magee Hospital of Pittsburgh.

---

Dr. Jacob Miller of Buffalo returned the middle of November from New York, where he took a course in the Polyclinic.

---

Dr. Wm. H. Heath of Buffalo has accepted a position as sanitarian for Siam. He will leave in January to assume his new duties to which his former service in the U. S. Marine Hospital Service (now Public Health Service)) and his long connection with the Buffalo Department of Health have so well fitted him.

